# *Drosophila* Avoids Parasitoids by Sensing Their Semiochemicals via a Dedicated Olfactory Circuit

**DOI:** 10.1371/journal.pbio.1002318

**Published:** 2015-12-16

**Authors:** Shimaa A. M. Ebrahim, Hany K. M. Dweck, Johannes Stökl, John E. Hofferberth, Federica Trona, Kerstin Weniger, Jürgen Rybak, Yoichi Seki, Marcus C. Stensmyr, Silke Sachse, Bill S. Hansson, Markus Knaden

**Affiliations:** 1 Max Planck Institute for Chemical Ecology, Jena, Germany; 2 Institute of Zoology, University of Regensburg, Regensburg, Germany; 3 Department of Chemistry, Kenyon College, Gambier, Ohio, United States of America; 4 Laboratory of Cellular Neurobiology, School of Life Sciences, Tokyo University of Pharmacy and Life Sciences, Hachioji, Tokyo, Japan; 5 Department of Biology, Lund University, Lund, Sweden; University of Lausanne, SWITZERLAND

## Abstract

Detecting danger is one of the foremost tasks for a neural system. Larval parasitoids constitute clear danger to *Drosophila*, as up to 80% of fly larvae become parasitized in nature. We show that *Drosophila melanogaster* larvae and adults avoid sites smelling of the main parasitoid enemies, *Leptopilina* wasps. This avoidance is mediated via a highly specific olfactory sensory neuron (OSN) type. While the larval OSN expresses the olfactory receptor Or49a and is tuned to the *Leptopilina* odor iridomyrmecin, the adult expresses both Or49a and Or85f and in addition detects the wasp odors actinidine and nepetalactol. The information is transferred via projection neurons to a specific part of the lateral horn known to be involved in mediating avoidance. *Drosophila* has thus developed a dedicated circuit to detect a life-threatening enemy based on the smell of its semiochemicals. Such an enemy-detecting olfactory circuit has earlier only been characterized in mice and nematodes.

## Abstract 2

A dedicated olfactory circuit helps the fruit fly to implement avoidance behavior in response to semiochemicals produced by its main parasitoid enemy – wasps of the genus *Leptopilina*.

## Author Summary

Detecting danger is a fundamental task for an animal. Larval parasitoids constitute clear danger to the vinegar fly *D*. *melanogaster* as up to 80% of fly larvae become parasitized in nature. We show that *Drosophila* larvae crawl away from places that smell like the main parasitoid enemies, *Leptopilina* wasps. Furthermore, *Drosophila* adult females avoid laying eggs at those places. This avoidance is mediated via a highly specific olfactory sensory neuron type that is tuned to detect three odors of the parasitoid, including the wasps sex pheromone iridomyrmecin. We identify the neuron type, the receptors, and the odor ligands that mediate this behavior and also show that this neuronal system is both necessary and sufficient to govern the parasitoid avoidance behavior. We also find evidence that this odor-based *Leptopilina* wasp avoidance is conserved across several *Drosophila* species.

## Introduction

The olfactory system is tuned to detect cues important to survival and reproduction. One extremely important function is to detect danger [1]. For humans, the odor of smoke is a good example of such an important olfactory warning signal to which we are highly sensitive. Only in two cases, however, have olfactory circuits specifically detecting predator or pathogen odor been characterized regarding the involved olfactory receptor and the danger-derived ligand; the cat urine detection in mice [2] and the pathogen detection in the nematode *Caenorhabditis elegans* [3]. For most insects, olfaction is the primary sense. They use it to find and judge food and oviposition sites [4,5], mates [6,7], or competitors [8], but so far no circuitry has been shown to be involved in detecting life-threatening enemies.

A major cause of death in larvae of the vinegar fly *D*. *melanogaster* is to be injected with eggs from a parasitoid wasp. The eggs develop into parasitoid larvae, which consume the fly larva from the inside. In some wild subpopulations, up to 80% of the fly larvae are parasitized by different parasitoid wasp species, with *Leptopilina boulardi* and *L*. *heterotoma* being the most common ones [9,10]. There is thus a very good reason for fly larvae to avoid being parasitized and for female flies to avoid laying eggs where parasitoids are present. With this background, we investigated the reaction of larval and adult vinegar flies to the smell of parasitoids. Both larvae and ovipositing flies showed a clear avoidance behavior to otherwise attractive food and oviposition substrates after these had been perfumed with a *Leptopilina* parasitoid bouquet. We could also demonstrate that in adult flies, avoidance was mediated by the ab10B neuron coexpressing the so-far orphan receptors Or49a and Or85f. This in turn allowed us to identify (-)-iridomyrmecin—a defensive allomone and sex pheromone component of *Leptopilina* [11,12]—as the sole ligand for Or49a and two other parasitoid odorants ((*R)-*actinidine and several stereoisomers of nepetalactol) as ligands for Or85f. As the corresponding neuron in fly larvae only expresses Or49a but not Or85f, larval detection of parasitoids was found to be governed only by iridomyrmecin. When we activated or inactivated the neurons artificially, we could show that they are necessary and sufficient to govern parasitoid avoidance behavior.

Recent investigations revealed that the fly has also developed several other survival strategies to escape parasitoid pressure. Female flies prefer ethanol-rich oviposition sites after they have visually recognized parasitoids. As *D*. *melanogaster* larvae have higher ethanol tolerance than their parasitoids, the flies self-medicate their offspring [13]. Furthermore, seeing parasitoids leads to sharp decline in flies’ oviposition [14]. Finally, upon wasp attack, fly larvae respond with a specific rolling behavior that occasionally flips the attacker to the back [15] and is mediated by a multimodal circuit that includes mechanosensory as well as nociceptive pathways [16]. All these evolutionary adaptations show how important it is for the fly to escape its deadly enemies, the parasitoids.

## Results and Discussion

### Flies Detect and Avoid Parasitoid Odor

**Fig 1:**
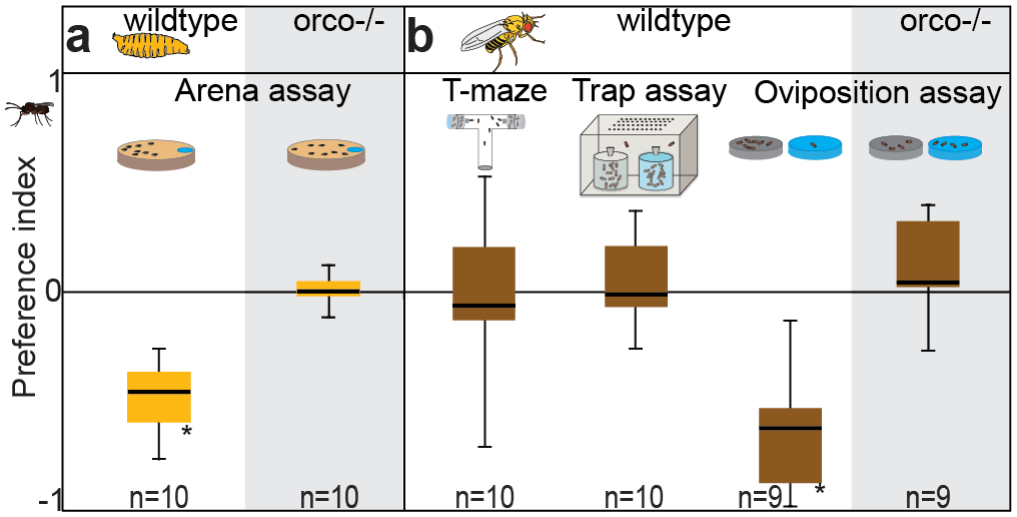
Larvae and ovipositing flies are repelled by parasitoid odor. (**A**) Larval choice assay and preference indices when larvae were exposed to the wash of *L*. *boulardi*. (**B**) Different choice assays (T-maze, Trap assay, Oviposition assay) for adult flies and resulting preference indices when exposed to the wash of *L*. *boulardi*. PI = (number of larvae, flies, or eggs in odor side − number in control side) / total number. Bar plots indicate minimum and maximum values (whiskers), the upper and lower quartiles (boxes) and the median values (bold black line). Deviation of the indices against zero was tested with Wilcoxon rank sum test.

We started our experiments with the main targets of the parasitoid and tested whether *Drosophila* larvae are repelled by the odor of parasitoid wasps (*L*. *boulardi*). Larvae were strongly repelled by the body wash of parasitoids (Fig 1A, for the behavioral data all figures are based on, see S1 Data). *Orco* mutant larvae, however, lacking functional odorant receptors (ORs), were not repelled (Fig 1A, grey shaded area), indicating that the avoidance behavior is elicited by volatile cues detected by ORs. We next examined the behavior in adult flies. In T-maze and trap assays, we did not observe any avoidance of parasitoid odor (Fig 1B). However, female flies strongly avoided the body wash odor when choosing an oviposition site (Fig 1B). Again, *Orco* mutant females lacked this avoidance (Fig 1B, grey shaded area). We conclude that the smell of *L*. *boulardi* is repellent to *D*. *melanogaster* larvae and ovipositing flies, and that this repellency is mediated by ORs. Obviously, the decision to oviposit seems to be governed by a circuit that is not directly involved in mediating attraction in adult flies—a finding that is in accordance with previous studies showing that both acetic acid [17] and limonene [18] are mediating oviposition but do not attract flies. As the parasitoids do not attack adult flies but only larvae, a general avoidance behavior of adult flies—apart from oviposition avoidance—does not seem to be adaptive.

**Fig 2:**
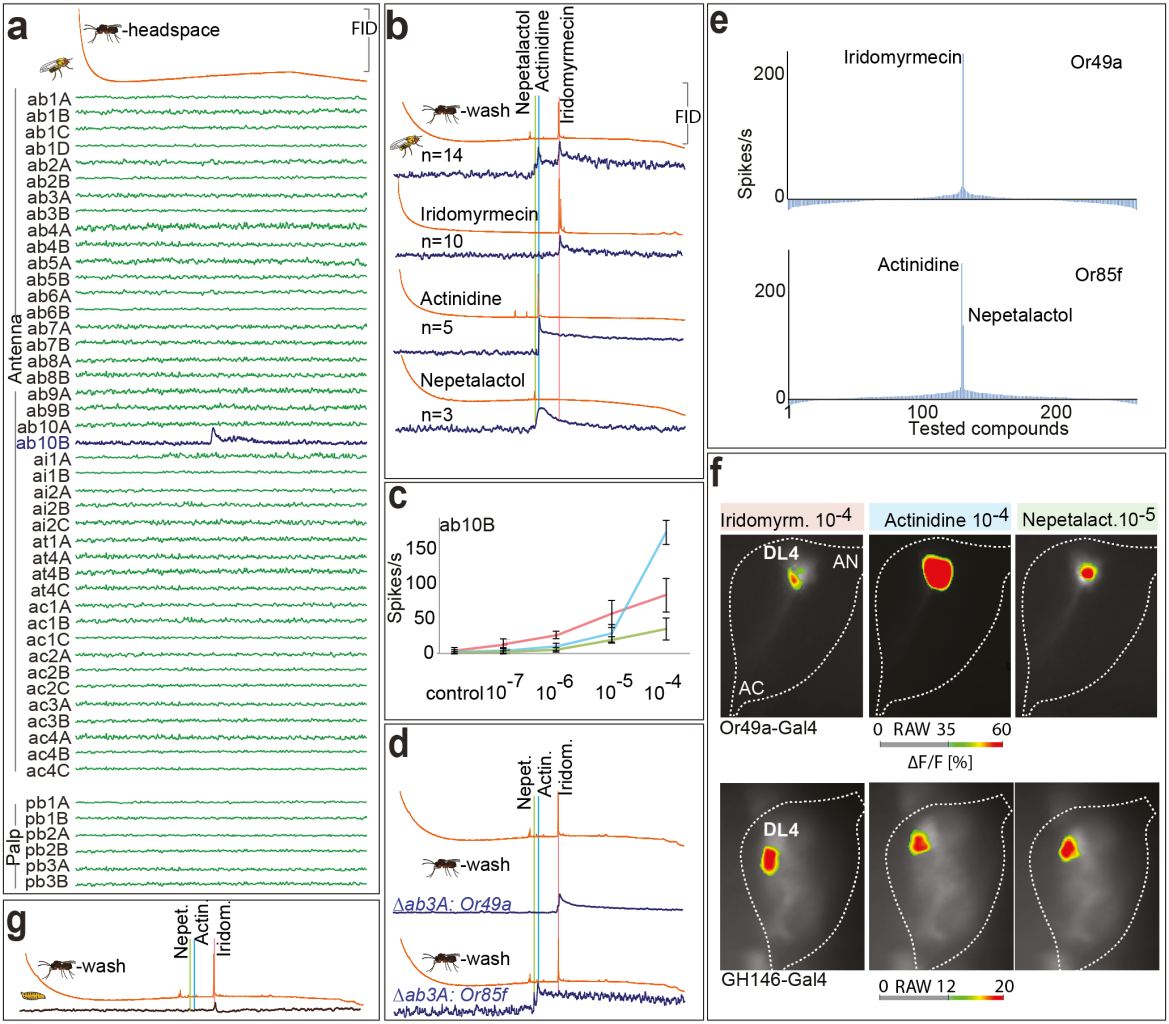
The ab10B neuron detects parasitoid odors. (**A**) Example spike traces of GC-coupled SSR with all *D*. *melanogaster* OSN types and the headspace of *L*. *boulardi* (note that the amount of odors within headspace is too low to be detected and analyzed by GC, but is still detected by ab10B). FID, flame ionization detector. (**B**) GC-coupled SSR with the ab10B neuron and the wash of *L*. *boulardi* (1st panel), as well as the identified active compounds (2nd–4th panel). (**C**) SSR dose-response curves of the ab10B neuron tested with active compounds. (**D**) GC-coupled SSR with mutant ab3A neuron ectopically expressing either Or49a or Or85f. Blue, green, and red lines indicate active compounds. (**E**) Tuning breadths of Or49a and Or85f. 232 odorants are displayed along the *x*-axis according to strengths of responses they elicit from each receptor. Odorants eliciting strongest responses are placed near the center of distribution. Negative values indicate inhibitory responses. For a list of compounds, see S4 Fig; for raw data see S1 Data. (**F**) Identification of glomeruli activated by parasitoid odors (-)-iridomyrmecin, (*R*)-actinidine, and nepetalactol (a mixture of 1S4aR7R7aS, 1R4aS7S7aS-nepetalactol and their enantiomers). 1st to 3rd columns, false color-coded images showing odorant-induced calcium-dependent fluorescence changes in OSNs expressing Or49a or PNs labeled by GH-146-Gal4 at the antennal lobe (AL) level. Flies express UAS-GCaMP3.0 under control of either Or49a-Gal4, or the GH146-Gal4 driver line. (**G**) GC-coupled extracellular recordings from larval dorsal organ and wash of *L*. *boulardi*. (for more GC-SSR traces of wildtype ab10B neurons and mutant ab3A neurons expressing Or49a or Or85f see S3 Fig)

We next identified the olfactory sensory neuron(s) (OSN) involved in detecting the parasitoid smell. *Drosophila* larvae express 23 different ORs, of which 13 are also expressed in the adult fly [19]. The identification of individual OSN responses in larvae is almost impossible, as all OSNs are colocalized in a single morphological structure, the dorsal organ. However, recording from identified adult OSNs is possible [20–24]. We therefore used a set of diagnostic odors (S1 Fig) to identify adult OSN types and afterwards performed combined gas chromatography-single sensillum recording (GC-SSR) experiments with the headspace of *L*. *boulardi*. We tested all 48 physiologically different OSN types present on the fly antennae and palps. Although the amount of active odor within the headspace was too low to be visible in the GC trace, a repeatable and strong response was elicited from the ab10B OSN at a specific GC retention time. No other OSN type responded to the extract (Fig 2A). To identify the active compound, we collected a larger quantity of odors by washing wasps in dichloromethane. When repeating the GC-SSR experiments with this wash, the ab10B OSN became activated at the same retention time as found with the headspace (Fig 2B), but now with a visible GC peak. In addition, the same OSN responded to two additional compounds.

Using mass spectrometry, we identified the three active peaks as a non-identified isomer of nepetalactol, (*R*)-actinidine, and (-)-iridomyrmecin (S2 Fig) and confirmed the identification by repeating GC-SSR experiments using synthetic standards (Fig 2B). Furthermore, dose-response experiments revealed that the ab10B neuron exhibited the highest sensitivity to iridomyrmecin (Fig 2C).

Nepetalactol is one of the major compounds of the volatile oil of catnip [25] and has been shown to be an insect repellent [26]. As an insect allomone, however, so far it has only been described for aphids [27]. Both actinidine and iridomyrmecin have been shown to be released by parasitoids. Actinidine acts as a defense compound [28], while iridomyrmecin, which was first described as a defensive compound of the Argentine ant, *Linepithema humilis* [29] fulfills many functions in the parasitoid wasps of the genus *Leptopilina* [11]. They use iridomyrmecin to defend themselves against predators, to avoid competition among females, and as a major component of the female sex pheromone [11,12]. As a consequence of its ubiquitary use in the chemical communication of *Leptopilina*, the wasps constantly release at least small amounts of iridomyrmecin, which makes it an ideal key substance to reveal the presence of these parasitoids to their host *Drosophila*.

As the ab10B OSN responds to these iridoids, and as it is one of the cases where two ORs (Or49a and Or85f) are coexpressed within the same OSN type in the adult fly [23], we next explored the role of the individual receptors in parasitoid odor detection. We selectively expressed either Or49a or Or85f ectopically in a *Drosophila* mutant ab3A neuron (i.e., a neuron lacking its own receptor Or22a [30]) and again performed GC-SSR experiments with the body wash of *L*. *boulardi*. OSNs expressing Or49a responded to iridomyrmecin exclusively (Fig 2D). Of the 16 possible stereoisomers of iridomyrmecin, only 3 (with (-)-iridomyrmecin being the most abundant) have been described to occur in the genus *Leptopilina* [11,12]. When testing those, we found that OSNs expressing Or49a responded only to (-)-iridomyrmecin, but not to (+)-iridomyrmecin or (+)- or (-)-isoiridomyrmecin (S5 Fig).

When we tested OSNs misexpressing Or85f, they became activated by (*R*)-actinidine and nepetalactol (Fig 2D). Further investigation revealed that the ab10B neuron detected both enantiomers of actinidine (although only (*R*)-actinidine is present in *Leptopilina* wasps). Because of chromatographic limitations, we were unable to determine the absolute configuration of the nepetalactol stereoisomer produced by the wasps. However, all synthetically available stereoisomers (i.e., 1S4aR7R7aS-nepetalactol, 1R4aS7S7aS-nepetalactol and their enantiomers) activated OSNs expressing Or85f (S6 Fig). Thus, both ORs expressed in the ab10B OSN are involved in the detection of the parasitoid volatile blend although sensitive to different components thereof. To further test the specificity of the ab10B OSN, we screened neurons ectopically expressing one of both receptors with a set of 232 compounds including odorants from a wide range of different chemical classes (Fig 2E, S4 Fig). From these results and from another study, where Or49a was tested against almost 500 odorants [31], we conclude that the ab10B neuron is highly specific to the *Leptopilina* odorants (-)-iridomyrmecin (Or49a), and (*R*)-actinidine and nepetalactol (Or85f). The spectrum of detection by the ab10B OSN has thus been widened, not by lessening the specificity of a single receptor but by adding a second highly specific one.

We next measured odor-induced activity patterns in the *D*. *melanogaster* adult antennal lobe (AL) (Fig 2F). We used the Gal4-UAS system to express the Ca^2+^-sensitive reporter GCaMP3.0 [32] under control of the Or49a promoter. Some of the former studies [23,33–36] did not observe expression of Or49a. However, we expressed GCaMP3.0 under control of *Or49a-Gal4* and validated the functionality of our *Or49a-Gal4* on the adult antenna using confocal microscopy (S7 Fig). All three parasitoid compounds elicited a strong activation. When we used the GH146 enhancer trap line, all three compounds elicited a clear and exclusive activation at the output level of the DL4 glomerulus. Hence, the calcium imaging results confirmed the conclusion from the SSR experiments that the ab10B neurons expressing Or49a and Or85f become activated by the parasitoid odorants (*R*)-actinidine, several enantiomers of nepetalactol, and (-)-iridomyrmecin. They also showed that these signals transfer via dedicated projection neurons to (PNs) higher brain centers. Recent findings suggest that laterally situated glomeruli of the AL mainly become activated by aversive stimuli [37]. Our finding that the DL4 glomerulus becomes activated by odorants governing parasitoid avoidance further supports this segregated representation of positive and negative innate olfactory valence within the medial and lateral AL, respectively.

Interestingly, in contrast to adult flies, larvae do not express Or85f but only Or49a [19]. As expected, when performing GC-coupled extracellular recordings from the dorsal organ with the body wash of parasitoids, we did not observe any responses to the Or85f ligands actinidine and nepetalactol, but observed a strong response to the Or49a ligand iridomyrmecin (Fig 2G). Contrary to adult females, larvae do not only lose part of their offspring but become killed by the parasitoid. Hence, the larval olfactory system should even be better suited to detect parasitoid odorants. It therefore remains unclear why *Drosophila* larvae do not express Or85f, and by that are restricted to detect iridomyrmecin only.

### Parasitoid Odor Activates Avoidance-Specific Brain Region

**Fig 3:**
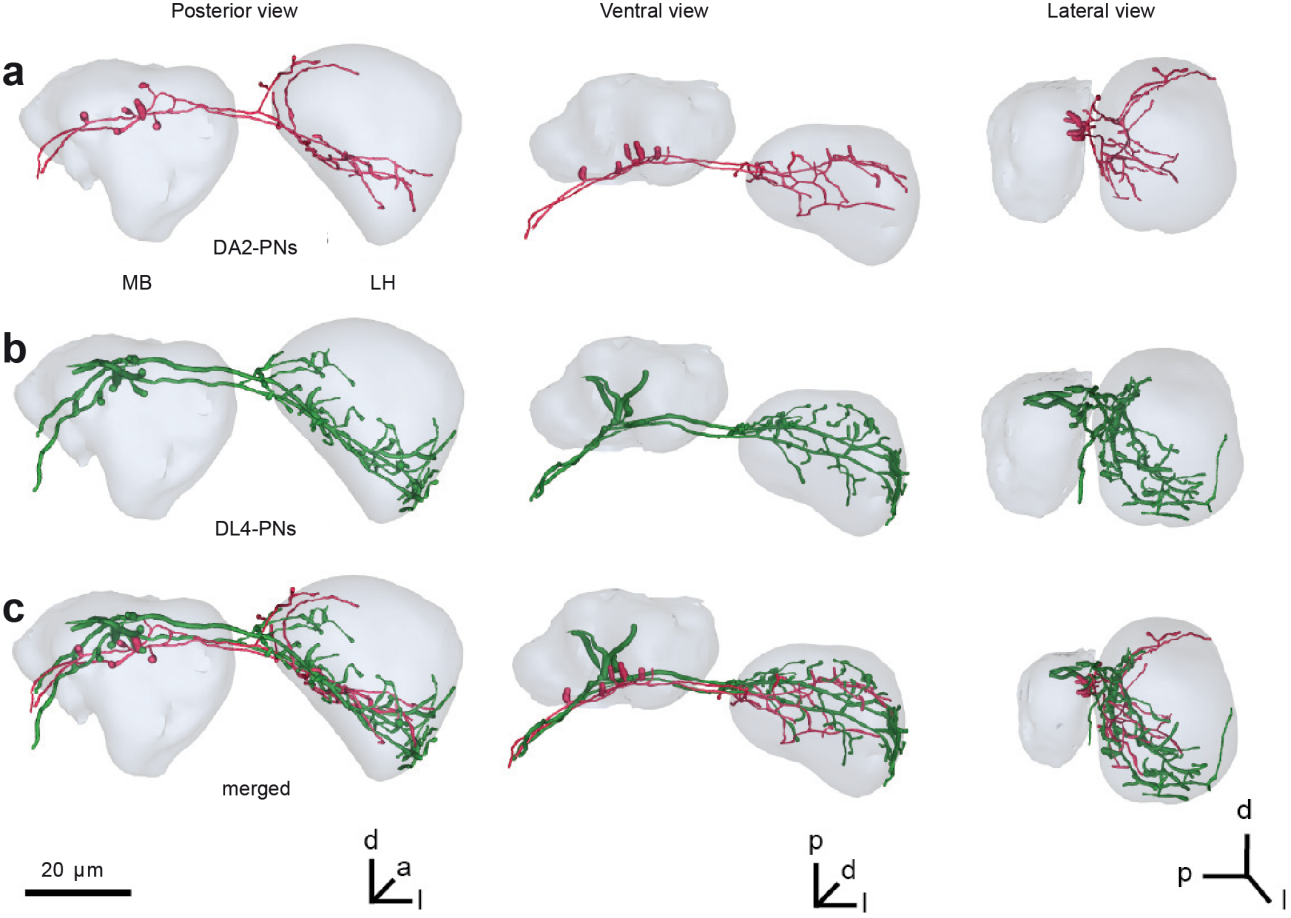
Innervation patterns of DL4 and DA2 PNs in MB and LH. (**A**) Reconstruction of two DA2 PNs. (**B**) Reconstruction of two DL4 PNs. (**C**) comparison of DA2 and DL4 domains after registration of datasets into a common reference space. DA2 and DL4 PNs overlap in the base of the MB and ventroposterior LH. a: anterior, d: dorsal, l: lateral, p: posterior v: ventral.

We next investigated how the information transfers to the next levels of olfactory processing, the mushroom body (MB) calyx and the lateral horn (LH). When characterizing the innervation patterns of DL4 PNs we found that the projection pattern of these neurons in higher brain centers was stereotypic among individual flies as shown for other PNs in previous studies [38,39]. We next compared how this innervation pattern relates to that of DA2-PNs conveying information from another highly repellent olfactory pathway mediating odor information regarding the presence of detrimental microbes [40]. Interestingly, the comparison revealed that the projection pattern of both PN types overlapped strongly within a posteriomedial domain in the LH and in the base MB calyx (Fig 3). The overlapping pattern of two PN populations that both convey information on odorants of strongly negative hedonic valence is in accordance with recent findings that olfactory stimuli with negative and positive valence activate separate domains in the LH [41–43].

### Importance of ab10B Neurons for Parasitoid Avoidance

We next tested whether the presence of one of the ab10B ligands was sufficient to inhibit oviposition. Flies exhibited a strong tendency to avoid the Or85f ligand (*R*)-actinidine (we applied 1 μg, i.e., ca 50 wasp equivalents) and oviposited significantly less when confronted with 3 wasp equivalents (i.e., 1 μg) of the Or49A ligand (-)-iridomyrmecin (S8 Fig). Furthermore, larvae only expressing Or49a consequently became significantly repelled by the Or49a ligand (-)-iridomyrmecin but did not avoid the Or85f ligand (*R*)-actinidine (S8 Fig).

**Fig 4:**
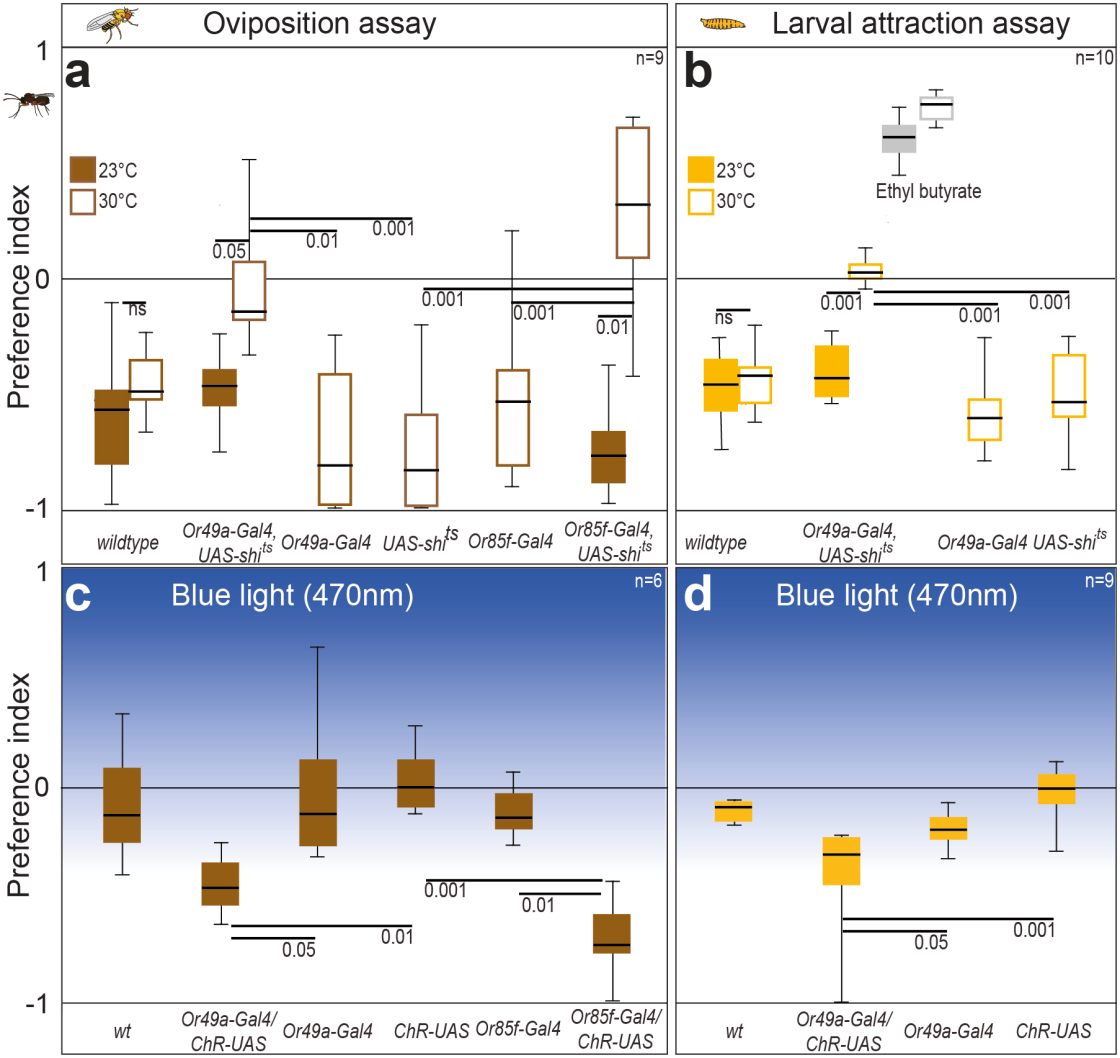
The ab10B neuron is necessary and sufficient to govern oviposition avoidance and larval avoidance behavior in *D*. *melanogaster*. (**A**) Preference indices of ovipositing wildtype flies, flies expressing *Shibire* ^*ts*^ in ab10B neuron, and corresponding parental lines at restrictive (30°C) and permissive (23°C) temperature when tested with wash of *L*. *boulardi*. (**B**) Preference indices of the same fly lines when tested in the larval assay. Attraction to ethyl butyrate (grey bars) depict that loss of odor-guided behavior in larvae expressing *Shibire* ^*ts*^ in ab10B neuron is odorant specific. (**C**) Light preference of ovipositing wildtype flies, flies expressing channelrhodopsin in ab10B neuron, and corresponding parental lines. (**D**) Light preferences of the same fly lines when tested in the larval assay. (**A–D**) Bar plots indicate minimum and maximum values (whiskers), the upper and lower quartiles (boxes), and the median values (bold black line). Groups were compared by the Kruskal Wallis test with a Dunn’s multiple comparison for selected pairs. For calculation of preference indices, see Fig 1.

Do ovipositing flies lacking a functional ab10B OSN still avoid the parasitoid smell? To silence the ab10B neurons specifically, we expressed the temperature-sensitive mutant dynamin Shibire^ts^ [44] under the control of the Or49a promoter. At the restrictive temperature (30°C), flies carrying this construct did not display any oviposition avoidance toward the parasitoid wash (Fig 4A), while they showed a strong aversion to the wash at the permissive temperature (23°C). Parental lines and wildtype flies avoided the parasitoid odor at the restrictive temperature. As Or49a and Or85f are coexpressed in the ab10B neuron, expressing Shibire^ts^ under the control of the Or49a promotor should erase the functional significance of both receptors. However, we also expressed Shibire^ts^ under the control of the Or85f receptor, which again yielded in reduced olfactory avoidance, when flies were tested at the restrictive temperature (Fig 4A).

Larvae that expressed Shibire^ts^ under the Or49a promoter again exhibited no avoidance at the restrictive temperature, while the permissive temperature resulted in avoidance not different from wild type and parental lines (Fig 4B). As the larvae at the restrictive temperature still targeted the attractant ethyl butyrate, the loss of behavior at restrictive temperature seems to be ligand- and Or49a-specific. We thus conclude that activation of OSNs expressing Or49a in larvae and Or49a and Or85f in adult flies is necessary for behavioral avoidance of parasitoid odors.

To test whether the activation of ab10B neurons was sufficient for avoidance of parasitoid smell, we expressed the photo-activated cation-selective channel channelrhodopsin-2 (*ChR2*) in the neurons by using either the *Or49a-Gal4* or the *Or85f-Gal4* driver. We then tested how blue light (470 nm, i.e., the wave length activating the expressed cation channel) affected adult oviposition and larval crawling behavior. Contrary to wild-type flies and parental lines, *CHR2-*females avoided illuminated oviposition sources (Fig 4C). The same held true for *CHR2-*larvae that became significantly more repelled by blue light than control lines (Fig 4D).

We conclude that activation of the ab10B neuron and its larval equivalent is necessary and sufficient to elicit avoidance of parasitoid smell in female *D*. *melanogaster* adults and larvae, respectively.

### ab10B Response Is Selective for *Leptopilina* Odor

**Fig 5:**
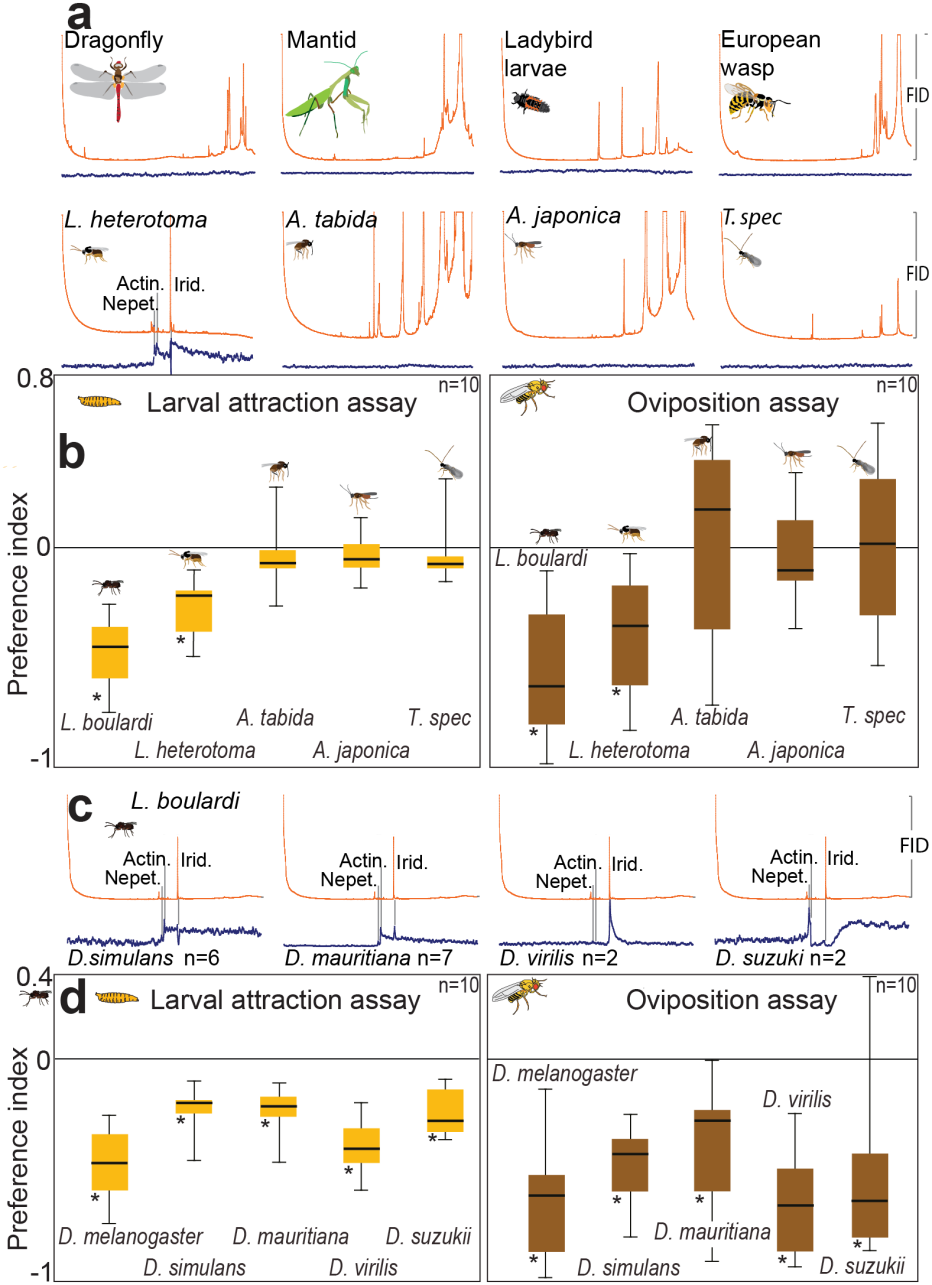
The ab10B neuron detects *Leptopilina* odors only, and detection and avoidance of Leptopilina odor are conserved within many drosophilid flies. (**A**) Example spike traces of GC-coupled SSR recordings of *D*. *melanogaster* ab10B neurons with washes of four potential predators (top row) and four parasitoid wasp species (bottom row). (**B**) Preference indices of *D*. *melanogaster* larvae and ovipositing females when tested with washes of five parasitoid species. Note that only washes of *L*. *boulardi* and *L*. *heterotoma* contain ligands of the ab10B neuron and provoked avoidance behavior in *D*. *melanogaster*. (**C**) Example spike traces of GC-coupled SSR recordings of ab10B neurons of 5 different *Drosophila* species with washes of *L*. *boulardi*. (**D**) Preference indices of larvae and ovipositing females of 5 *Drosophila* species tested with wash of *L*. *boulardi*. (**A–B**) Deviations of indices against zero were tested with Wilcoxon rank sum test. Asterisks, *p* < 0.05; error bars depict standard deviation.

*D*. *melanogaster* becomes parasitized not only by *Leptopilina* wasps but also by other wasp genera. We asked whether the ab10B neuron also responded to odorants from predators or other parasitoid species (Fig 5A). Therefore, we performed GC-SSR experiments with ab10B neurons using body washes of four potential *Drosophila* predators and of four additional parasitoid wasp species (*L*. *heterotoma*, *Asobara tabida*, *A*. *japonica*, and *Trichopria* spec.). While the neurons again responded to iridomyrmecin, actinidine, and nepetalactol present in *L*. *heterotoma*, we could not identify any further ab10B ligand—neither in the odor of the insect predators nor in that of the other parasitoid species. Accordingly, *D*. *melanogaster* avoided the odor of *L*. *boulardi* and *L*. *heterotoma* but neither ovipositing adult flies nor larvae displayed any odor-based avoidance behavior (Fig 5B) towards the predators or the parasitoids belonging to other genera. We conclude that the ab10B-dependent avoidance behavior is restricted to one of the most harmful parasitoid genera [9,10,45], the iridoid-producing *Leptopilina*.

### Avoidance of *Leptopilina* Odor Is Not Restricted to *D*. *melanogaster*

Finally, we asked whether odor-based detection and avoidance of *Leptopilina* wasps is specific for *D*. *melanogaster*. Parasitoids of the genus *Leptopilina* do not only target *D*. *melanogaster* but also many other *Drosophila* species [46,47]. All of the 12 *Drosophila* species, whose genomes have been published, express Or49a and Or85f. However, the orthologs of Or49a (Or85f) only show 57.1% (63.5%) amino acid similarity with a dN/dS value of 0.23 (0.19), respectively. We therefore asked whether detection and avoidance can also be observed in *D*. *simulans*, *D*. *mauritania*, *D*. *virilis*, and *D*. *suzukii*. As all four species either originate from Africa or Asia, i.e., continents where wasps of the genus *Leptopilina* also occur, they should all have a long evolutionary history with at least one *Leptopilina* species. In GC-SSR experiments with each of the *Drosophila* species, we always found an OSN that responded to the headspace of *L*. *boulardi* (Fig 5C). We cannot exclude that the OSN responding to wasp odors in those flies expressed another so-far unidentified receptor. However, in all species, the colocalized neurons exhibited similar responses like the *D*. *melanogaster* ab10A neuron (S9 Fig, for raw data see S1 Data). Therefore—also in the other species—it was most probably the ab10 sensillum that detected the wasp odor, which supports the general involvement of Or49a and Or85f in parasitoid detection of the genus *Drosophila*. Furthermore, larvae and ovipositing adults of all tested species avoided the parasitoid headspace (Fig 5D and 5E). We conclude that iridoid-based *Leptopilina* avoidance is a conserved feature of several *Drosophila* species. Whether or not the detection of the *Leptopilina* odors is mediated in all species by Or49a and Or85f remains open. However, the comparatively low conservation of these receptors is not necessarily a counterargument. It has been shown before that receptors exhibiting low sequence conservation in different *Drosophila* species still can be tuned to the same ligands [48]. Another functionally conserved receptor, Or56a, on the other hand is also one of the molecularly most conserved receptors among the drosophilids. Evolution obviously finds different ways to preserve function among olfactory receptors.

### Conclusion

We show that drosophilid flies and their larvae avoid the odor of one genus of parasitoids. The question, why *Drosophila* exhibits a dedicated circuit to the odors of *Leptopilina* wasps while the odors of the other tested parasitoid wasps did not evoke any olfactory avoidance behavior at all, remains open. Although *Leptopilina* seems in some habitats to be the most abundant parasitoid, parasitation rates by other genera can become high [9,10,45]. However, *Drosophila* does not only exhibit olfactory parasitoid avoidance but also avoidance based on visual cues [13,14] as well as on mechanosensory and nociceptive cues [15]. It, therefore, might be that the nonolfactory cues become especially important in the avoidance of those parasitoids that do not carry any of the ab10B ligands. The animals tested in our experiments had never experienced parasitoids before. Hence, the observed avoidance behavior is governed by innate rather than learned negative hedonic valence. Dedicated pathways involved in detection and processing of innate valence are usually restricted to odorants of outstanding ecological valence [49] and are not restricted to insects [50]. Zebrafish detect the death-associated odor cadaverine based on the specific and highly sensitive receptor TAAR13c [51]. Mice detect predators like cats, snakes, and rats [52] as well as conspecifics [53] based on olfactory cues. It has been shown that the trace amine-associated receptor 4 (TAAR4) mediates innate repellence to cat urine [2], while TAAR5 detects a compound in mouse urine and mediates attraction towards conspecifics [53]. Recent findings in *Drosophila* revealed several dedicated olfactory pathways that either mediate avoidance [40,54], mating [55], or oviposition [18]. Enemy avoidance based on learned cues have been shown for *C*. *elegans*. After first contact with the pathogen *Serratia marcescens*, the nematode learns to avoid the bacterium based on a specific bacterium-derived peptide [3]. We show that the fly’s ab10B neuron specifically mediates innate aversion towards the parasitoid-derived odorants (-)-iridomyrmecin, (*R*)-actinidine and different enantiomers of nepetalactol. To our knowledge, this presents the first case in which an animal not only smells and avoids its enemy but does this based on the enemy’s semiochemicals, including a sex pheromone. The evolutionary strategy to use the odor-based sex communication system of an enemy to avoid it should be highly adaptive. The possibility for evolutionary countermeasures from the parasitoid side should be limited as it is difficult to resign the sex communication system.

## Methods

### Experimental Procedures

#### *Drosophila* stocks

All experiments with wild type (WT) *D*. *melanogaster* were carried out with the Canton-S strain. Species other than *D*. *melanogaster* were obtained from the *Drosophila* species stock center (https://stockcenter.ucsd.edu/info/welcome.php). Transgenic lines were obtained from the Bloomington *Drosophila* stock center (http://flystocks.bio.indiana.edu/), except for the *w* ^*118*^; Δ*halo/cyo; UAS-Or49a*, *w* ^*118*^, and Δ*halo/cyo; UAS-Or85f*, which were a kind gift from Dr. J.R. Carlson (Yale University, USA), and w[*]; P{w [+ mC] = UAS − ChR2.S}3, which was a kind gift from Dr. Andrè Fiala (Georg August University, Germany).

#### Rearing of wasps

We used *D*. *melanogaster* as the host to rear *L*. *boulardi*. To rear a cohort of *L*. *boulardi*, 20–30 *Drosophila* flies of mixed age and sex were put into a jar containing an approximately 2 cm thick layer of standard corn based rearing medium. After 48 h, the flies were removed and 5 to 10 mated females of *L*. *boulardi* were put in the jar. Wasps emerge approximately 28 d after oviposition and were kept at 25°C, 60% humidity and a 16:8 h light:dark cycle. For experiments, we either used the headspace of 20 parasitoid wasps or the body wash of 100 parasitoid wasps (solved in 1,000 μl of dichloromethane).

#### T-maze assays

T-maze experiments were carried out as described in [40]. In brief, 30 4–5 d-old female flies were introduced into the bottom part of a t-shaped tube (length of each arm, 4 cm; diameter, 1 cm) and over 40 min were allowed to enter (but not to leave) via pipette tips (tip opening, 2 mm) eppendorff caps attached to the two upper arms of the t-shaped tube. The lids of the Eppendorff caps contained 0.5 ml agar (1%). In addition, each Eppendorff cap contained a piece of filter paper that was loaded either with 50 μl of dichloromethane containing the equivalent of the wash of 3 wasps or with solvent only. We let the solvent evaporate for 5–10 min before the filter papers were added to the caps. The attraction index (AI) was calculated as AI = (O − C) / 30, where O is the number of flies entered the odorant containing trap and C is the number of flies entered the solvent containing trap.

#### Trap assays

Trap assay experiments were performed as described in [37]. In brief, 50 4–5 d old female flies were introduced in to a small box (length, 10 cm; width, 8 cm; height, 10 cm) that contained two smaller containers (height, 4.5 cm; diameter, 3 cm). For 24 hr, flies could enter (but not leave) these containers through a pipette tip (tip opening, 2 mm). Containers were equipped with the lid of an eppendorff cap that was loaded either with 50 μl of dichloromethane containing the equivalent of the wash of 5 wasps or with solvent only. The attraction index (AI) was calculated as AI = (O − C) / 50, where O is the number of flies entered the odorant containing trap, and C is the number of flies that entered the solvent-containing trap.

#### Oviposition assays

Oviposition assay experiments were carried out in a cage (50 x 50 x 50 cm) that was equipped with two petri dishes (diameter, 9 cm) containing agar (1%), of which one was loaded with 50 μl of dichloromethane containing the equivalent of the wash of 5 wasps or with solvent only. Thirty 4–5 d-old female flies were placed in each cage. Experiments were carried out in a climate chamber (25°C, 70% humidity, 12 h light:12 h dark cycle). The number of eggs was counted after 24 hr. Oviposition index was calculated as (O − C) / (O + C), where O is the number of eggs on a baited plate, and C is the number of eggs on a control plate.

#### Larval two-choice assays

The larval olfactory choice assay was performed as described in [56]. Briefly, 50 second or third instar larvae were placed in the center of a Petri dish, filled with 1% agrose. The Petri dish contained two discs of filter paper (diameter, 0.5 cm) placed at opposite positions at the periphery of the dish. Filter papers were loaded either with 20 μl of dichloromethane containing the equivalent of the wash of 2 wasps or with 20 μl of dichloromethane only. Larvae were allowed to crawl for 5 min before their position on the Petri dish was determined. Attraction index was calculated as ((O − C) / T), with O being the number of larvae on the side of the dish loaded with wasp odor, C being the number of larvae on the solvent side, and T being the total number of larvae.

#### Shibire experiments

The experiments with larvae or flies expressing shibire^ts^ were performed as described above except that the temperature was set as either 23°C (permissive temperature) or at 30°C (restrictive temperature).

#### Channelrhodopsin experiments

For channelrhodopsin-2 (ChR2) experiments, larvae or adult flies were raised in darkness on food inoculated with 200 μl of 150 mM all-trans retinal (Sigma, Germany). Oviposition and larval experiments were performed in a petri dish, filled with 1% agarose, which contained a single LED-emitting blue light (480 nm wavelength) on one side, while the other side was not illuminated. In the larval experiment, 50 larvae were allowed to crawl for 5 min before their position on the Petri dish was determined. Attraction index was calculated as ((O − C) / T), with O being the number of larvae on the illuminated side of the dish, C being the number of larvae on the nonilluminated side, and T being the total number of larvae. The oviposition experiment with flies expressing channelrhodopsin-2 was slightly modified from the oviposition assay mentioned above. A single Petri dish (illuminated on one side by a blue LED) was located in a small container (length, 10 cm; width, 10 cm; height, 10 cm) that prohibited the desiccation of the illuminated (and hence slightly heated) agar. After 24 hr, eggs on the illuminated and the nonilluminated side were counted. Attraction index was calculated as ((O − C) / T), with O being the number of eggs on the illuminated side of the dish, C being the number of eggs on the nonilluminated side, and T being the total number of eggs.

### SSR/GC-SSR

Adult flies were immobilized in pipette tips, and the third antennal segment or the palps were placed in a stable position onto a glass coverslip. Sensilla were localized under a binocular at 1,000x magnification and the extracellular signals originating from the OSNs were measured by inserting a tungsten wire electrode in the base of a sensillum. The reference electrode was inserted into the eye. Signals were amplified (10x; Syntech Universal AC/DC Probe, www.syntech.nl), sampled (10,667 samples/s), and filtered (100–3,000 Hz with 50–60 Hz suppression) via USB-IDAC connection to a computer (Syntech). Action potentials were extracted using Syntech Auto Spike 32 software. We used a set of diagnostic odors (S1 Fig, i.e., odors that have been shown to activate neurons expressing a specific receptor [20]) to identify adult OSN types. As in *D*. *melanogaster*, the colocalization of neurons expressing different receptors in a single sensillum is conserved; we were able to identify even those neurons with orphan receptors by checking out the response patterns of colocalized neurons. Neuron activities were recorded for 10 s, starting 2 s before a stimulation period of 0.5 s. Responses from individual neurons were calculated as the increase (or decrease) in the action potential frequency (spikes/s) relative to the prestimulus frequency. For GC stimulation, 1 μl of the odor sample (1 μl of actinidine and nepetalactol solutions correspond to ca 50 wasp equivalents, 1 μl of iridomyrmecin solution corresponds to 3 wasp equivalents, 1 μl of wasp wash corresponds to 0.1 wasp equivalent) was injected onto a DB5 column (Agilent Technologies, http://www.agilent.com), fitted in an Agilent 6890 GC, equipped with a four-arm effluent splitter (Gerstel, www.gerstel.com), and operated as previously described (Stökl et al., 2010) except for the temperature increase, which was set at 15°C min^-1^. GC-separated components were introduced into a humidified airstream (200 ml min^-1^) directed toward a mounted fly. Signals from OSNs and FID were recorded simultaneously.

### Chemical Analysis

Solvent extract of 100 wasps were analysed by gas chromatography coupled with mass spectrometry (GC-MS). Samples were analysed on a Shimadzu GC2010 gas-chromatograph (GC) connected to a QP2010 plus mass-spectrometer (MS; Shimadzu, Germany) and an Agilent 7890GC coupled with an 5975c MS (Agilent Technologies, Germany). The Shimadzu GC-MS was equipped with either a nonpolar capillary column (BPX-5, 30 m length, 0.25 mm inner diameter, 0.25 μm film thickness; SGE Analytical Science, UK), or a BetaDex 225 cyclodextrin column (30 m length, 0.25 mm inner diameter, 0.25 μm film thickness; Sigma-Aldrich, Germany) or a GammDex 120 cyclodextrin column (30 m length, 0.25 mm inner diameter, 0.25 μm film thickness; Sigma-Aldrich, Germany). When using the BPX5 column, Helium was used as a carrier gas with a constant linear velocity of 50 cm/s^-1^ and the temperature program of the GC oven started at 80°C and was raised by 5°C/min^-1^ to 280°C. For both cyclodextrin columns, the carrier gas flow was reduced the 35 cm/s^-1^ and the oven temperature started at 50°C and was raised by 2°C/min^-1^ to 220°C. The MS was run in electron impact (EI) mode at 70 eV and set to a scan range from 35 to 600 mz^-1^. All samples were injected splitless. The Agilent GC-MS was equipped with a Cyclosil-B cyclodextrin column (30 m length, 0.25 mm inner diameter, 0.25 μm film thickness; Agilent Technologies, Germany). Helium was used as a carrier gas with a constant linear velocity of 36 cm/s^-1^. The initial temperature of the GC oven of 40°C was held for 2 min and afterwards raised by 2°C/min^-1^ to 170°C and then with 70°C to 250°C. The MS was run in electron impact (EI) mode at 70 eV and set to a scan range from 33 to 350 mz^-1^. All samples were injected splitless. Compounds were identified by comparing the mass spectrum and retention time with that of synthetic reference compounds (S2 Fig).

### Synthetic Compounds

Stereoisomers of Iridomyrmecin were synthesized as described in [12] and [57]. Actinidine and Nepetalactol were synthesized as described by [58].

### Optical Imaging

Flies were prepared for optical imaging as previously described by [59]. Briefly, flies were anesthetized on ice; the head capsule was opened by incising the cuticle between the antennae and the eyes. With the brain immersed in Ringer’s saline (130 mM NaCl, 5 mM KCl, 2 mM MgCl2, 2 mM CaCl2, 36 mM sucrose, 5 mM Hepes, [pH 7.3]), the ALs were exposed by removing muscle tissue, glands, and the trachea. We used a Till Photonics imaging system with an upright Olympus microscope (BX51WI) equipped with a 20x Olympus objective (XLUM Plan FL 20x/0.95W). A Polychrome V provided light excitation (475 nm) and a filter set ensured passage of only relevant wavelengths (excitation: SP500, dicroic: DCLP490, emission: LP515). The emitted light was captured by a CCD camera (Sensicam QE, PCO AG) with a symmetrical binning of 2 (0.625 x 0.625 μm/pixel). For each measurement, a series of 40 frames was taken (4 Hz). Odors were applied for 2 sec. Pure compounds were diluted in mineral oil (Carl Roth GmbH + Co. KG); 6 μl of the diluted odors were pipetted onto a small piece of filter paper (~1 cm^2^, Whatman), placed inside a glass Pasteur pipette. Filter papers were prepared ca. 30 min before every experimental session. For odor application, a stimulus controller (CS-55, Syntech) provided a continuous air flow (l/min) in which odor injection was applied via two disposable Pasteur pipettes. For odor stimulation, the air stream switched from a blind Pasteur pipette to the stimulus pipette. Calcium imaging recordings were processed using custom-written software in IDL (ITT Visual Information Solutions) as described in detail in [59]. An in vivo 3-D atlas of the *Drosophila* AL [60] served to link the calcium signals to identified glomeruli.

### Registration of uPNs into Reference Space in Central Brain

Neurons were intracellulary stained with biocytin or Lucifer yellow and histologically processed as described previously [40,61]. For brain neuropil background staining, the synaptic antibody nc82 [62] was used. High-resolution scans were done with a Zeiss LSM 510 confocal microscope using a 63 x water immersion objective. Image stacks were imported to Amira 5.6, and neuronal arborizations and neuropil were manually segmented. For neuron reconstruction, the Skeleton plugin [63] of Amira was used. For registration of datasets, a label template of the central brain (MB and LH) was chosen out of *n* = 58 preparations following the method of [64]. The warping of segmented labels of the MB and LH onto the template was done in a two-step process: an affine transformation with 12 degree of freedom (12 DOF) followed by an elastic registration using modules of Amira. The calculated transformation matrix was applied to the neuron reconstruction and thus transformed to the template reference space.

## Supporting Information

S1 Data Raw data the figures of this manuscript are based on. (XLSX)

S1 Fig Diagnostic set of odors used to identify OSNs during SSRs. OSNs that are expected to exhibit strong responses to a specific odor are given in brackets.
(TIF)

S2 Fig Iridoid compounds produced by *L*. *boulardi* and *L*. *heterotoma*. Total ion current (TIC) chromatograms on a nonpolar (BPX5) GC column of an extract of females of (**A**) *L*. *boulardi* and (**B**) *L*. *heterotoma*. (**C**) Molecular structure of the iridoid compounds found in *L*. *boulardi* and *L*. *heterotoma*. Numbers correspond to the peaks in (A) and (B). (**D–G**) Identification of (*R*)-actinidine: (**D**) TIC chromatograms on a cyclodextrin (CycloSil B) GC column of synthetic (*S*)- and (*R*)-actinidine, and extracts of *L*. *boulardi* and *L*. *heterotoma*. (**E**) Mass spectrum of synthetic (*R*)-actinidine and the indicated peak in *L*. *boulardi* (**F**) and *L*. *heterotoma* (**G**). The peaks found in the extracts of *L*. *boulardi* and *L*. *heterotoma* show the same retention time and mass spectrum as (*R*)-actinidine.
Identification of nepetalactol (**H–M**): (**H**) TIC chromatograms on a cyclodextrin (CycloSil B) GC column of racemic samples of synthetic 1R4aS7S7aS- and 1S4aR7R7aS-nepetalactol. The dashed lines indicate the peaks of nepetalactol. (**I**) and (**J**) mass spectra of the first peak in 1R4aS7S7aS- and 1S4aR7R7aS-nepetalactol, respectively. (**K**) TIC chromatograms of extracts of *L*. *boulardi* and *L*. *heterotoma* and the mass spectrum of the indicated peak in *L*. *boulardi* (**L**) and *L*. *heterotoma* (**M**). The peaks in the extracts of *L*. *boulardi* and *L*. *heterotoma* show the same retention time and mass spectrum as nepetalactol. The four stereoisomers of nepetalactol available as authentic standards could not be separated on any of the three cyclodextrin column tested. Therefore, the absolute configuration of the nepetalactol produced by the wasps remains unknown.
The color of the mass spectra corresponds to the color of the chromatograms in the same row.
(TIF)

S3 Fig Electrophysiological recordings with *Drosophila* OSNs and wasp odours. **(A–D)** SSR responses of wildtype ab10B neurons tested with the headspace **(A)** or wash **(B)** of *L*. *boulardi*, or synthetic (-)-iridomyrmecin **(C),** (*R*)-actinidine **(D),** or nepetelactol (a mixture of 1S4aR7R7aS-Nepetalactol, 1R4aS7S7aS-Nepetalactol and their enantiomers) **(E)**. **(F)** Dorsal-organ recordings of wildtype larvae tested with the bodywash of *L*. *boulardi*. **(G–H)** SSR responses of mutant ab3A neuron-expressing Or49a **(G)** or Or85f **(H)** tested with bodywash of *L*. *boulardi*.
(TIF)

S4 Fig Odor panel used to screen Or49a and Or85f in the empty neuron system, color-coded by functional group (red, alcohols; blue, esters; gray, acids; brown, ketones; pink, aldehydes; light green, nitrogen-containing compounds; purple, terpenes; dark green, alkanes; black, other compounds). (TIF)

S5 Fig GC-SSR responses of a neuron misexpressing Or49a to different isomers of iridomyrmecin. Top line named with the compound depicts the flame ionization detector (FID) signal of the GC.
(TIF)

S6 Fig GC-SSR responses of a neuron misexpressing Or85f to different isomers of actinidine and nepetalactol. Top line named with the compound depicts the FID signal of the GC.
(TIF)

S7 Fig Localization of OSNs expressing Or49a on the antenna of a female *D*. *melanogaster*. OSNs are visualized by expressing GCaMP3.0 under control of *Gal4*-*Or49a* driver line.
(TIF)

S8 Fig Behavioral avoidance of synthetic compounds. Larval choice assay and oviposition assay and resulting preference indices when exposed to the synthetic (-)-iridomyrmecin and (*R*)-actinidine. Deviation of the indices against zero was tested with Wilcoxon rank sum test. Asterisks, *p* < 0.05; error bars depict standard deviation. PI = (number of larvae, flies, or eggs in odor side − number in control side) / total number.
(TIF)

S9 Fig Response profiles of neurons paired with iridomyrmecin-, actinidine-, and nepetalactol -responsive neurons shown in Fig 4C (*n* = 3). Error bars represent standard error of the mean (SEM).
(TIF)

## Abbreviations

AL: antennal lobe
FID: flame ionization detector
GC-SSR: gas chromatography-single sensillum recording
LH: lateral horn
MB: mushroom body
OR: odorant receptor
OSN: olfactory sensory neuron
PN: projection neuron
SEM: standard error of the mean
TAAR: trace amine-associated receptor
TIC: total ion current
