# Supplementary figures and images for: Drosophila Avoids Parasitoids by Sensing Their Semiochemicals via a Dedicated Olfactory Circuit
Source: PLoS Biol. 2015 Dec 16;13(12):e1002318. doi: 10.1371/journal.pbio.1002318 (PMC4687525; doi:10.1371/journal.pbio.1002318)

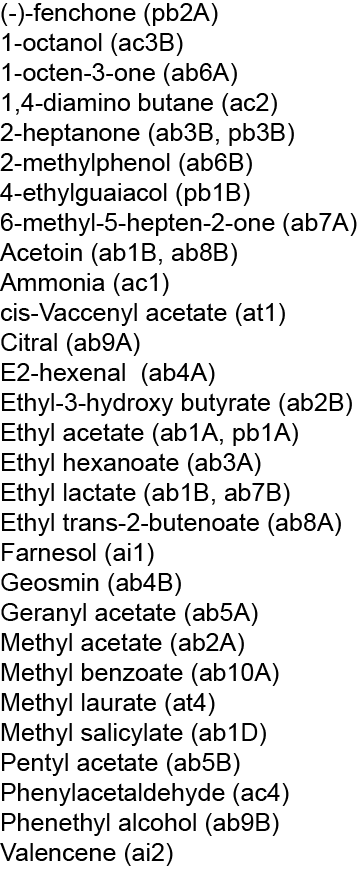

Supplement: S1 Fig — OSNs that are expected to exhibit strong responses to a specific odor are given in brackets. (TIF) [file pbio.1002318.s002.tif]

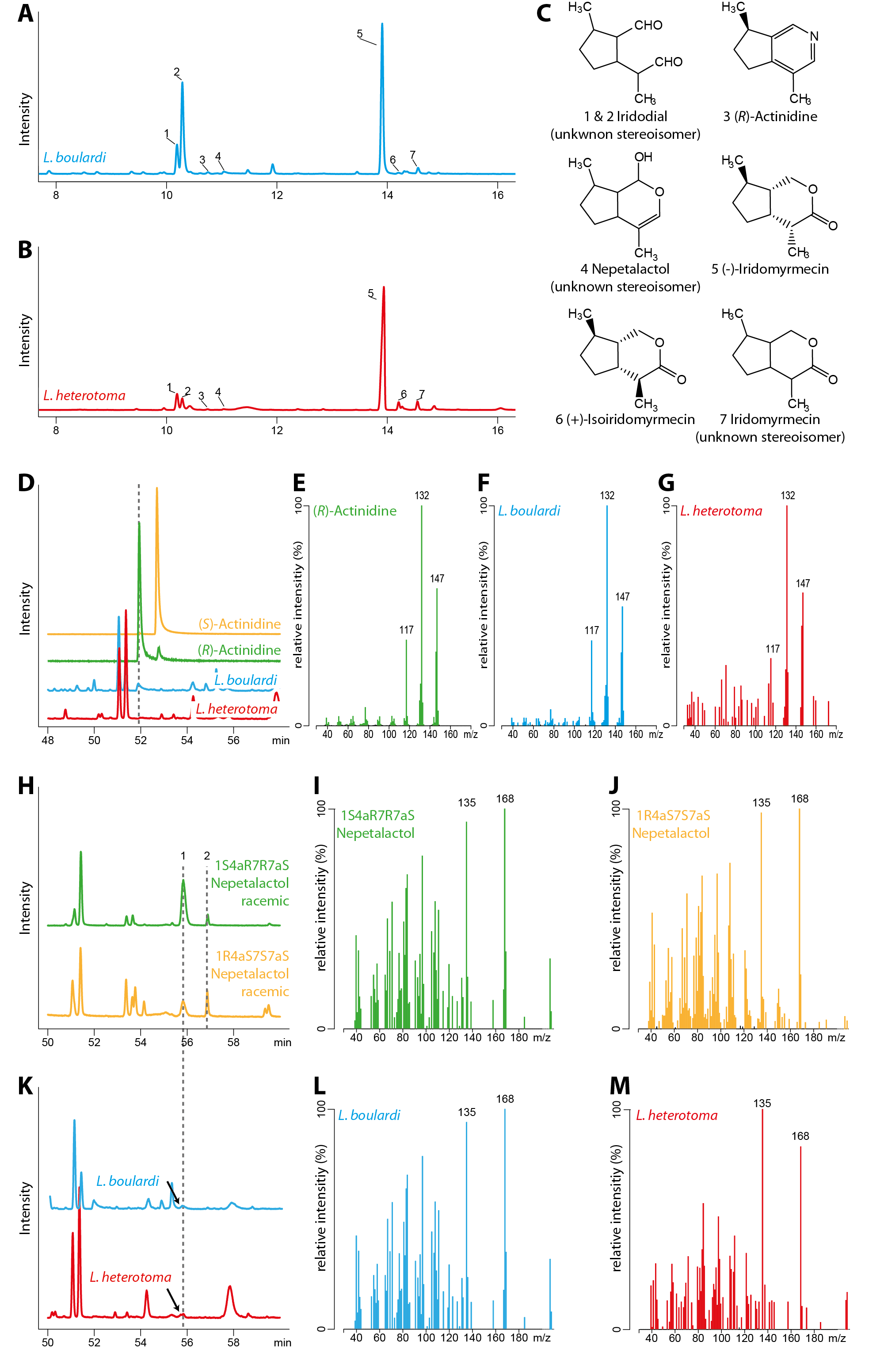

Supplement: S2 Fig — Total ion current (TIC) chromatograms on a nonpolar (BPX5) GC column of an extract of females of (A) L. boulardi and (B) L. heterotoma. (C) Molecular structure of the iridoid compounds found in L. boulardi and L. heterotoma. Numbers correspond to the peaks in (A) and (B). (D–G) Identification of (R)-actinidine: (D) TIC chromatograms on a cyclodextrin (CycloSil B) GC column of synthetic (S)- and (R)-actinidine, and extracts of L. boulardi and L. heterotoma. (E) Mass spectrum of synthetic (R)-actinidine and the indicated peak in L. boulardi (F) and L. heterotoma (G). The peaks found in the extracts of L. boulardi and L. heterotoma show the same retention time and mass spectrum as (R)-actinidine. Identification of nepetalactol (H–M): (H) TIC chromatograms on a cyclodextrin (CycloSil B) GC column of racemic samples of synthetic 1R4aS7S7aS- and 1S4aR7R7aS-nepetalactol. The dashed lines indicate the peaks of nepetalactol. (I) and (J) mass spectra of the first peak in 1R4aS7S7aS- and 1S4aR7R7aS-nepetalactol, respectively. (K) TIC chromatograms of extracts of L. boulardi and L. heterotoma and the mass spectrum of the indicated peak in L. boulardi (L) and L. heterotoma (M). The peaks in the extracts of L. boulardi and L. heterotoma show the same retention time and mass spectrum as nepetalactol. The four stereoisomers of nepetalactol available as authentic standards could not be separated on any of the three cyclodextrin column tested. Therefore, the absolute configuration of the nepetalactol produced by the wasps remains unknown. The color of the mass spectra corresponds to the color of the chromatograms in the same row. (TIF) [file pbio.1002318.s003.tif]

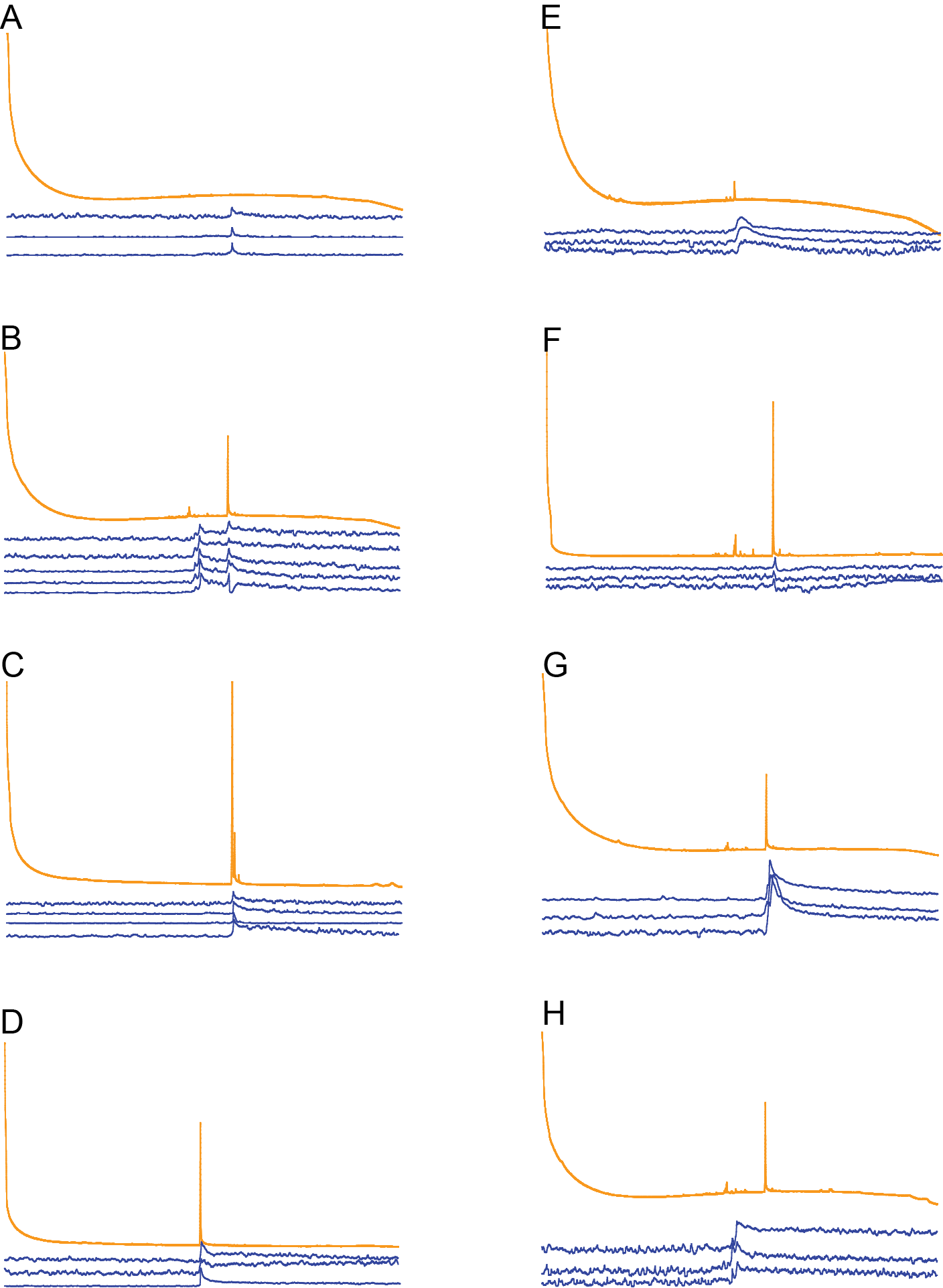

Supplement: S3 Fig — (A–D) SSR responses of wildtype ab10B neurons tested with the headspace (A) or wash (B) of L. boulardi, or synthetic (-)-iridomyrmecin (C), (R)-actinidine (D), or nepetelactol (a mixture of 1S4aR7R7aS-Nepetalactol, 1R4aS7S7aS-Nepetalactol and their enantiomers) (E). (F) Dorsal-organ recordings of wildtype larvae tested with the bodywash of L. boulardi. (G–H) SSR responses of mutant ab3A neuron-expressing Or49a (G) or Or85f (H) tested with bodywash of L. boulardi. (TIF) [file pbio.1002318.s004.tif]

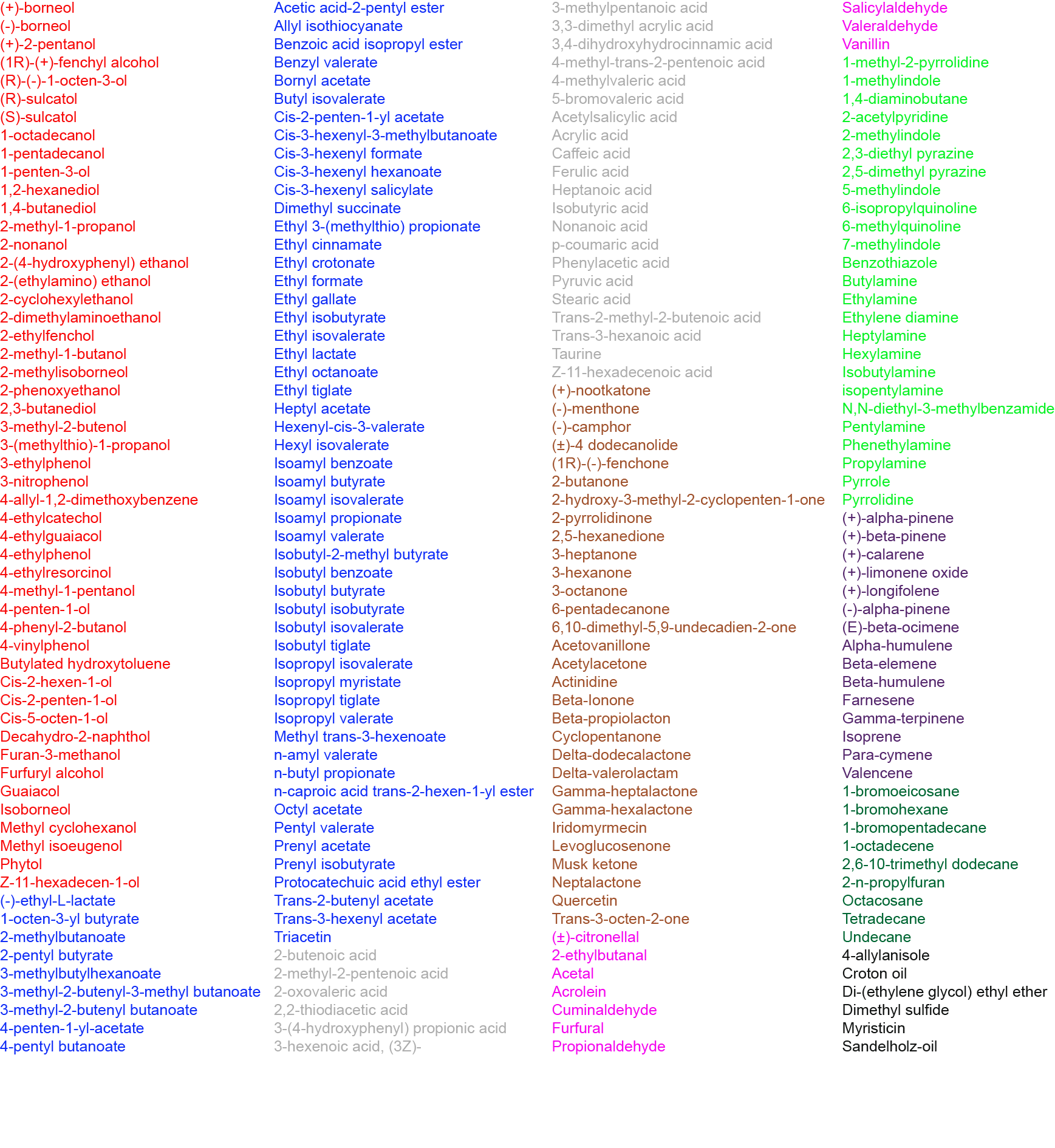

Supplement: S4 Fig — (TIF) [file pbio.1002318.s005.tif]

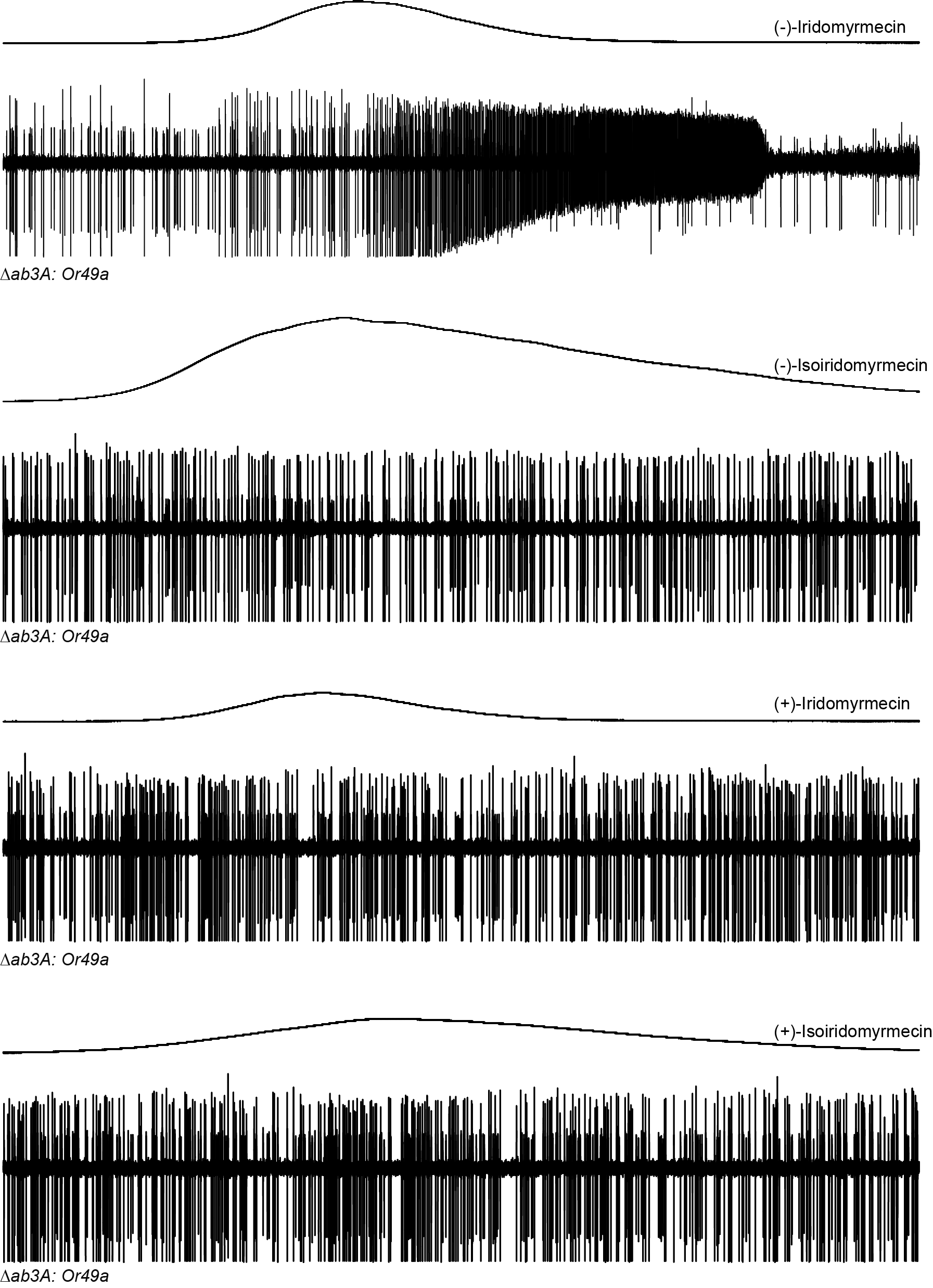

Supplement: S5 Fig — Top line named with the compound depicts the flame ionization detector (FID) signal of the GC. (TIF) [file pbio.1002318.s006.tif]

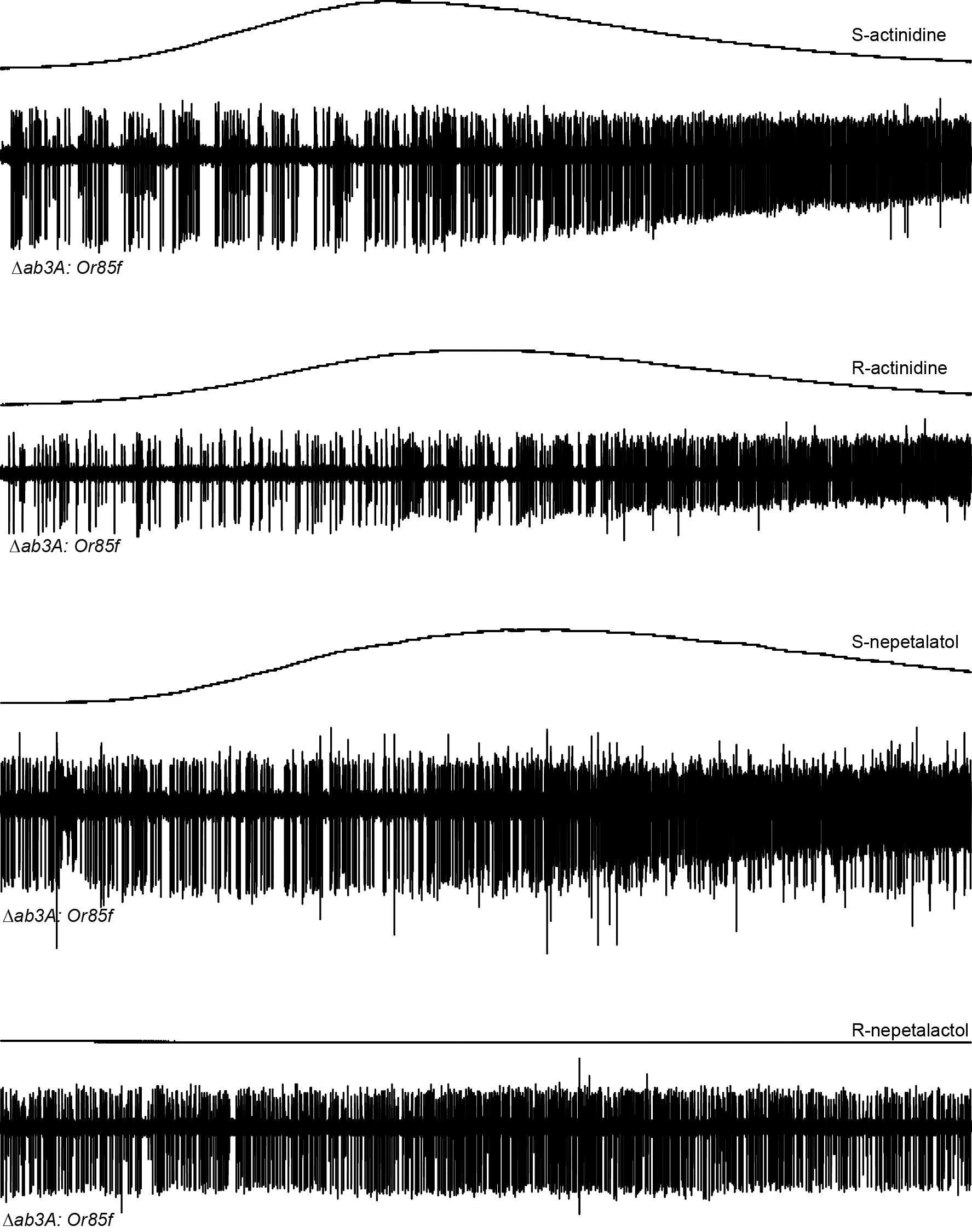

Supplement: S6 Fig — Top line named with the compound depicts the FID signal of the GC. (TIF) [file pbio.1002318.s007.tif]

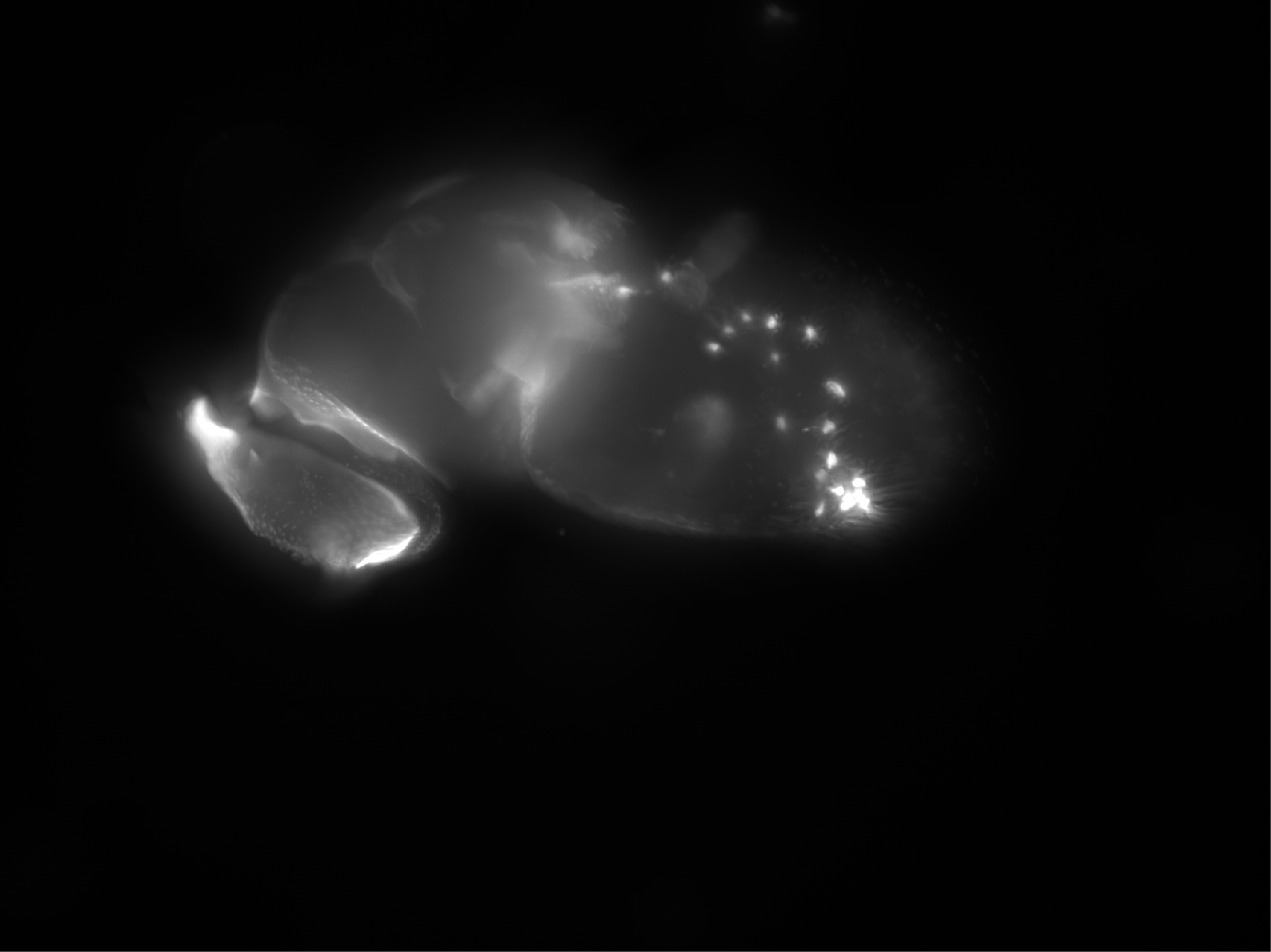

Supplement: S7 Fig — OSNs are visualized by expressing GCaMP3.0 under control of Gal4-Or49a driver line. (TIF) [file pbio.1002318.s008.tif]

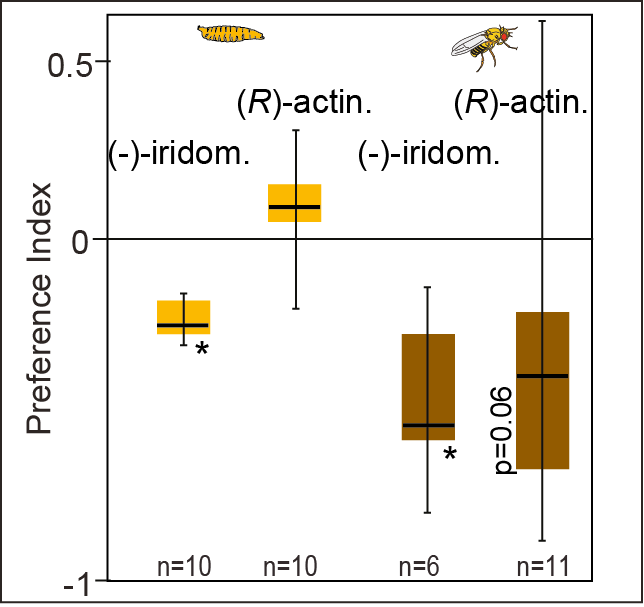

Supplement: S8 Fig — Larval choice assay and oviposition assay and resulting preference indices when exposed to the synthetic (-)-iridomyrmecin and (R)-actinidine. Deviation of the indices against zero was tested with Wilcoxon rank sum test. Asterisks, p < 0.05; error bars depict standard deviation. PI = (number of larvae, flies, or eggs in odor side − number in control side) / total number. (TIF) [file pbio.1002318.s009.tif]

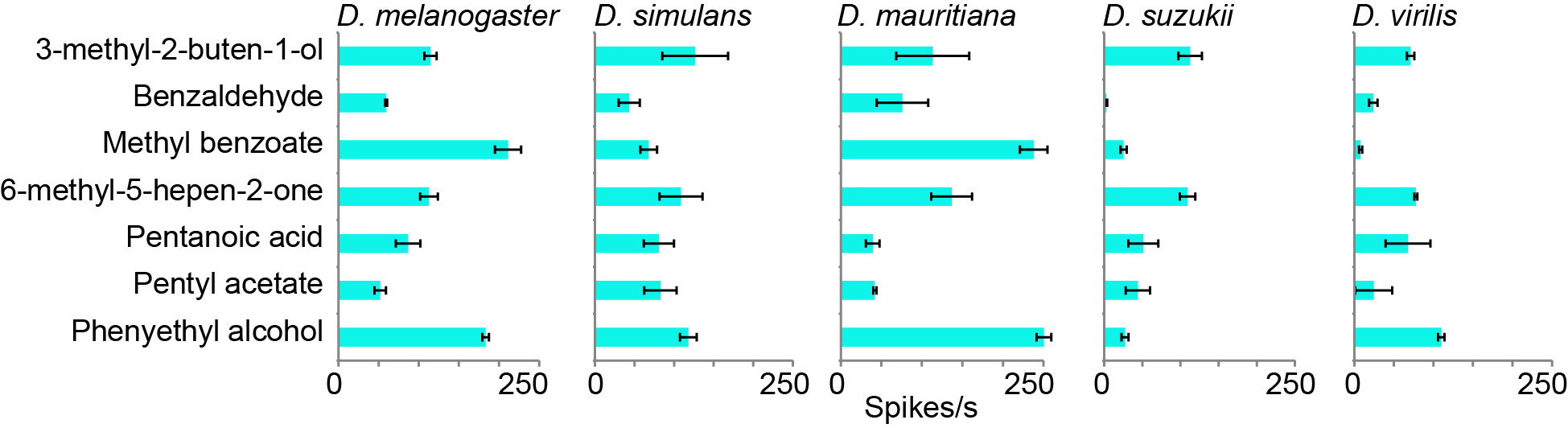

Supplement: S9 Fig — Error bars represent standard error of the mean (SEM). (TIF) [file pbio.1002318.s010.tif]
